# On Medical Evidence

**Published:** 1875-12

**Authors:** 


On Medical Evidence.—The eminent obstetrician, Dr. Robert Barnes, observes, in a recent lecture: "Seldom, probably, does a scientific witness leave the box quite easy in mind as to whether he has spoken 'the truth, the whole truth, and nothing but the truth;' and not seldom does he experience the mortification of finding what he has said utterly wrested from its proper meaning by the perversity of counsel, or misunderstood by the court and jury. The theory that through the conflict of opposing counsel, by examination and cross-examination, and the impartial interrogation of the court and jury, the truth is elicited, often signally fails in practice. The questions of counsel are generally prompted by hints gathered from books, or by experts sitting at their elbows. But they are very wary in accepting these suggestions. They may not understand them; and, if they do, they may, in their discretion, fear that the answer drawn will damage their case. Thus it constantly happens that the expert is only allowed to say so much of the truth as may suit the purpose of counsel; and every one knows how detached fragments of truth may be made to support a false argument.—*Med. and Surg. Reporter.*

